# Analysis of expressed sequence tags from a single wheat cultivar facilitates interpretation of tandem mass spectrometry data and discrimination of gamma gliadin proteins that may play different functional roles in flour

**DOI:** 10.1186/1471-2229-10-7

**Published:** 2010-01-11

**Authors:** Susan B Altenbach, William H Vensel, Frances M DuPont

**Affiliations:** 1 USDA-ARS Western Regional Research Center, 800 Buchanan Street, Albany, CA 94710 USA

## Abstract

**Background:**

The gamma gliadins are a complex group of proteins that together with other gluten proteins determine the functional properties of wheat flour. The proteins have unusually high levels of glutamine and proline and contain large regions of repetitive sequences. While most gamma gliadins are monomeric proteins containing eight conserved cysteine residues, some contain an additional cysteine residue that enables them to be linked with other gluten proteins into large polymers that are critical for flour quality. The ability to differentiate among the gamma gliadins is important for studies of wheat flour quality because proteins with similar sequences can have different effects on functional properties.

**Results:**

The complement of gamma gliadin genes expressed in the wheat cultivar Butte 86 was evaluated by analyzing publicly available expressed sequence tag (EST) data. Eleven contigs were assembled from 153 Butte 86 ESTs. Nine of the contigs encoded full-length proteins and four of the proteins contained nine cysteine residues. Only one of the encoded proteins was a perfect match with a sequence reported in NCBI. Contigs from four different publicly available EST assemblies encoded proteins that were perfect matches with some, but not all, of the Butte 86 gamma gliadins and the complement of identical proteins was different for each assembly. A specialized database that included the sequences of Butte 86 gamma gliadins was constructed for identification of flour proteins by tandem mass spectrometry (MS/MS). In a pilot experiment, proteins corresponding to six Butte 86 gamma gliadin contigs were distinguished by MS/MS, including one containing the extra cysteine residue. Two other proteins were identified as one of two closely related Butte 86 proteins but could not be distinguished unequivocally. Unique peptide tags specific for Butte 86 gamma gliadins are reported.

**Conclusions:**

Inclusion of cultivar-specific gamma gliadin sequences in databases maximizes the number and quality of peptide identifications and increases sequence coverage of these gamma gliadins by MS/MS. This approach makes it possible to distinguish closely related proteins, to associate individual proteins with sequences of specific genes, and to evaluate proteomic data in a biological context to better address questions about wheat flour quality.

## Background

The wheat gluten proteins represent about 80% of the grain protein and confer the unique viscoelastic properties that enable the production of bread, noodles, tortillas and many other food products from wheat flour. The gluten proteins are made up of gliadins, a heterogeneous collection of monomeric proteins that are associated with extensibility properties, and glutenins, a group of proteins that confer elasticity properties and form some of the largest polymers known in nature (reviewed by [1,2]). Both gliadins and glutenins have highly repetitive sequences with an abundance of glutamine and proline residues. The gliadins have molecular weights (MWs) from about 28,000 to 55,000 and are separated into alpha, gamma and omega subgroups, each containing many protein species with similar sequences. Proteins within each subgroup have distinct N-terminal and C-terminal sequences. The structures of the proteins and the number and arrangement of cysteine residues within proteins of each gliadin subgroup also are distinct. Alpha gliadins contain 6 conserved cysteine residues that form three intrachain crosslinks and gamma gliadins contain 8 conserved cysteine residues that form four intrachain crosslinks. Omega gliadins do not contain any cysteine. The glutenins consist of LMW-glutenin subunits with MWs similar to those of alpha and gamma gliadins and HMW-glutenin subunits of about 67,000 to 88,000 MW that are linked by interchain disulfide bonds into polymers that range from 500,000 to more than 10 million MW. Both the amount and the size of the glutenin polymers are important for flour quality. In addition to the traditional gliadins and glutenins, certain proteins with amino acid sequences very similar to gliadins contain an extra cysteine residue that enables them to be incorporated into the glutenin polymer [3]. These proteins are structurally similar to gliadins but functionally similar to LMW-glutenin subunits. It has been hypothesized that these proteins serve as chain terminators of the glutenin polymer thereby limiting its size and influencing the quality of the flour [4].

Environmental conditions during wheat grain development can affect wheat flour quality. Changes in the accumulation of individual gluten proteins during grain development in response to high temperatures and fertilizer have been detected in the US bread wheat Butte 86 [5,6]. However, it has been difficult to determine the identities of individual gluten proteins using approaches that work for most other proteins, in part because the gluten proteins yield relatively few peptides of a size suitable for MS/MS analysis when digested with trypsin, the enzyme used in most proteomic studies [7]. Additionally, the identification of proteins by MS/MS is based on the best fit of spectral data to databases of protein sequences. Although current databases contain sequences for many wheat gluten proteins, these represent only a portion of the sequence heterogeneity present in the gluten protein families. To distinguish individual gamma gliadin proteins from the US wheat Butte 86 that are influenced by temperature or fertilizer and identify gliadin-like proteins that could serve as chain terminators, we first examined the complement of gamma gliadin genes expressed in this cultivar by analyzing publicly available EST data. The availability of protein sequences derived from these genes allowed us to interpret MS/MS data obtained from an analysis of Butte 86 flour and associate individual gluten proteins with the sequences of specific genes.

## Results

### Assembly of Butte 86 contigs for gamma gliadins

A total of 153 ESTs from *Triticum aestivum *cv. Butte 86 developing grain were identified by searching the Dana Farber Cancer Institute *Triticum aestivum *Gene Index (TaGI) Release 10.0 [8]. One hundred forty one of these were assembled into new contigs (Table 1.) Accession numbers for ESTs within each contig and consensus sequences of contigs can be found in Additional Files 1 and 2, respectively. Twelve ESTs represented partial sequences of gamma gliadin genes that did not align with any other ESTs. These were excluded from further analysis (Additional File 1). Sixteen ESTs, all representing 3' portions of gamma gliadin coding regions, were assigned to more than one contig. Of these, seven ESTs were assigned to both contigs #1 and 8 and nine ESTs were assigned to both contigs #3 and 10 (Additional File 1). Although the 5' end of contig #8 is represented by only one EST, [GenBank: BQ805093], this EST has sufficient overlap (270 bp) with others to justify the creation of a separate contig. Similarly, one EST, [GenBank: BQ806189], represents the 5' end of contig #10. [GenBank: BQ806189] has more than 400 bp overlap with several ESTs that make up the 3' end, again justifying the creation of a separate contig.

**Table 1:** Best matches of consensus sequences from Butte 86 contigs to NCBI non-redundant database.

Contig #	# ESTs	NCBI Accession^1^	Identity	Source	Type
1	19^2^	[GenBank: AF234644.1]	1195/1227	*T. aestivum *cv. Cheyenne	mRNA
2	34	[GenBank: M16064.1]	1187/1200	*T. aestivum *cv. Yamhill	gene
3	18^3^	[GenBank: M36999.1]	1088/1094	*T. aestivum *cv. Yamhill	gene
4	14	[GenBank: FJ006605.1]	1005/1007	*T. aestivum *cv. Chinese Spring	gene
5	27	[GenBank: FJ006695.1]	1139/1191	*Aegilops speltoides*	gene
6	17	[GenBank: EF426565.1]	1048/1054	*T. turgidum *ssp. durum cv. Langdon	gene
7	4	[GenBank: M13713.1]	1083/1087	*T. aestivum *cv. Yamhill	gene
8	8^2^	[GenBank: AF234644.1]	1083/1123	*T. aestivum *cv. Cheyenne	mRNA
9^4^	3	[GenBank: FJ006688.1]	848/908	*Aegilops searsii*	gene
		[GenBank: FJ006686.1]	848/908	*Aegilops searsii*	gene
10	10^3^	[GenBank: M36999.1]	1024/1078	*T. aestivum *cv. Yamhill	gene
11^5^	3	[GenBank: EF151018.1]	668/668	*T. aestivum *cv. Chinese Spring	mRNA

^1 ^with best score by blastn against nr/nt database, no filters, no masks, last searched on 5/11/09.

^2 ^includes seven ESTs assigned to both contigs #1 and #8.

^3 ^includes nine ESTs assigned to both contigs #3 and 10.

^4 ^contig is missing a portion of the 3' end of coding region.

^5 ^contig is missing a portion of the 5' end of the coding region.

Contigs #2 and 5 contain the greatest number of ESTs, 34 and 27, respectively, suggesting that these sequences are the most highly expressed gamma gliadins in Butte 86 (Table 1). Contigs #1, 3, 4 and 6 contain between 9 and 17 ESTs not found in any other contigs, suggesting that these gamma gliadin genes are moderately expressed. Contigs #7, 9 and 11 are likely to be expressed at low levels since each contains only 3 or 4 ESTs. Contigs #8 and 10 also may be expressed at low levels, given that each contains only one EST not assigned to any other contig.

When used to search the NCBI non-redundant database, the consensus sequences of eight Butte 86 contigs (#1, 2, 3, 4, 7, 8, 10, 11) were most similar to DNA sequences identified in *T. aestivum*, one contig (#6) was most similar to a sequence from *T. turgidum *and two contigs (#5, 9) were most similar to sequences in *Aegilops *species (Table 1). Only contig #11 was a perfect match with a sequence reported previously.

### Comparison of Butte 86 gamma gliadin contigs with contigs from other EST assemblies

A number of EST databases include sequences from Butte 86 developing grain. However, contigs from these databases are not necessarily accurate representations of the gamma gliadins present in this cultivar. Each database includes a different collection of ESTs, utilizes different algorithms for assembly, and groups ESTs somewhat differently. To evaluate which EST databases were likely to contain the most accurate representation of Butte 86 gamma gliadin genes, contigs containing each Butte 86 gamma gliadin EST were identified in four publicly available EST assemblies (Additional File 1). The US Wheat Genome Project assembly [9,10] included 117,510 ESTs and 18,876 contigs. This assembly used Phrap with penalty-5, minmatch 50, minscore 100, thereby allowing any 2 sequences with at least 90% sequence similarity over 100 bases to form a contig. All ESTs from nine of the Butte 86 contigs (#1, 2, 3, 4, 5, 6, 7, 9, 11) were assigned to single contigs in the US Wheat Genome Project assembly, while ESTs from Butte 86 contigs #8 and 10 were distributed between two different contigs. The HarvEST:Wheat Version 1.14 assembly (WI all NSF "stringent" from 05/08/04) [11] contained 101,107 ESTs and 16,718 contigs. This assembly used CAP3 [12]. All ESTs from six Butte 86 contigs (#3, 5, 6, 7, 9,11) were found in single contigs in HarvEST 1.14, while ESTs from four contigs (#1, 2, 4, 10) were divided between two contigs and ESTs from contig #8 were distributed among three different contigs. TaGI assemblies from Dana Farber Cancer Institute [8] required a minimum match of 40 base pairs with greater than 94% identity in the overlap region and a maximum unmatched overhang of 30 base pairs. TaGI Release 10.0 contained 580,155 ESTs and 44,954 contigs. All of the ESTs contained within seven Butte 86 contigs (#2, 3, 4, 6, 7, 10, 11) were grouped in single contigs in TaGI Release 10.0. ESTs from Butte 86 contigs #5, 8 and 9 were found in two TaGI contigs while ESTs from contig #1 were distributed among three TaGI contigs. ESTs from only five Butte 86 contigs (#3, 5, 7, 9,11) were grouped in single contigs in the updated TaGI assembly, Release 11.0, which included 1,034,368 ESTs. ESTs from Butte 86 contigs #1, 2, 4 and 10 each fell into two contigs and ESTs from contigs #6 and 8 were each distributed among three contigs in this assembly.

### Characterization of Butte 86 gamma gliadin proteins

**Figure 1:**
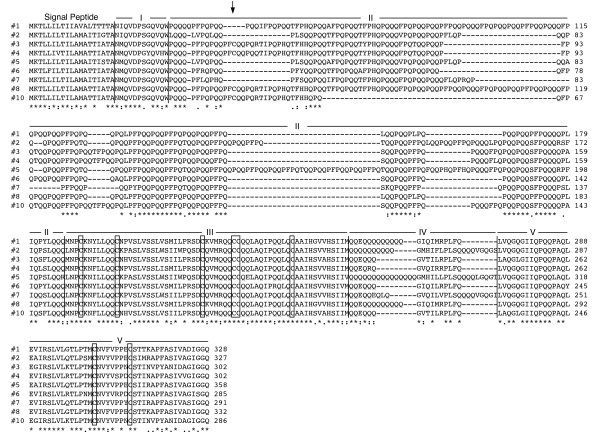
**Alignment of full-length gamma gliadins deduced from consensus sequences of Butte 86 contigs**. Alignments were performed with ClustalW2 using a blosum matrix and default settings. Identical residues are indicated by asterisks, conserved substitutions by colons and semi-conserved substitutions by periods. The eight conserved cysteine residues are enclosed in boxes. The position of the extra cysteine residue in #3, 4, 8 and 10 is indicated with an arrow.

Consensus sequences for nine of the eleven Butte 86 contigs contain complete coding regions for gamma gliadin proteins. Contig #9 is missing a portion of the 3' end of the coding region and contig #11 is missing a portion of the 5' end of the coding region (Table 1). Full-length proteins encoded by the nine contigs range from 285 to 358 amino acids (Table 2). All contain a 19 amino acid signal peptide predicted by the Signal P algorithm [13]. The predicted MWs of the mature proteins range from 30,361 to 38,899 and the predicted pIs of the proteins range from 7.5 to 8.1 (Table 2). Figure 1 shows a comparison of the amino acid sequences of the full-length gamma gliadins encoded by Butte 86 contigs. Protein domains typical of gamma gliadins are indicated as described by Anderson et al. [14]. Regions I, III and V are very similar in the different Butte 86 proteins and the positions of 6 cysteine residues in region III and 2 cysteine residues in region V are conserved. Regions II and IV vary among the proteins. Region II is comprised of between 114 and 174 amino acids in the nine Butte 86 gamma gliadins, encompassing from 42 to 51% of each mature protein sequence (Table 2). Proline and glutamine make up between 74 and 82% of the amino acid residues and determine the repetitive nature of this region. Four of the Butte 86 gamma gliadins (#3, 4, 8, 10) also contain an additional cysteine residue in this portion of the protein. Region IV ranges from 16 to 33 amino acids in length in the Butte 86 proteins with much of the difference due to strings of glutamine residues.

**Table 2:** Characteristics of full-length proteins encoded by Butte 86 contigs.

					Region II				# Immunogenic Peptides^1^			
							
Contig #	# amino acids^2^	# Cys	MW^3^	pI^3^	# amino acids	# Pro	# Gln		QQPQQPFPQ^4^	PQQPFPQQPQQ^5^	PQQSFPQQQ^6^	IIQPQQPAQ^7^
1	328	8	35481	7.5	156	50	78		6	2	0	1
2	327	8	35148	7.9	149	41	73		7	3	1	1
3	302	9	32294	7.5	136	38	63		4	0	1	1
4	302	9	32569	7.8	136	36	65		3	0	1	1
5	358	8	38899	7.6	174	51	84		9	4	1	0
6	285	8	30581	8.1	119	35	59		4	0	1	1
7	291	8	30975	7.9	114	31	54		2	0	1	1
8	332	9	36035	7.8	160	50	77		6	2	0	1
10	286	9	30361	7.5	120	34	56		4	0	1	1

^1 ^epitopes likely to play a role in celiac disease.

^2 ^including signal peptide.

^3 ^mature protein.

^4 ^described by Qiao et al. [15].

^5 ^described by Molberg et al. [16].

^6 ^described by Sjostrom et al. [17].

^7 ^described by Arentz-Hansen et al. [18].

The gamma gliadins from Butte 86 differ in their complements of epitopes that are likely to be involved in celiac disease (Table 2). While all of the full-length Butte 86 gamma gliadins contain multiple copies of the QQPQQPFPQ epitope defined by Qiao et al. [15], only gamma gliadins #1, 2, 5 and 8 contain the PQQPFPQQPQQ sequence defined by Molberg et al. [16]. All but gamma gliadins #1 and 8 contain a single copy of the PQQSFPQQQ epitope reported by Sjostrom et al. [17] and all but gamma gliadin #5 contain single copies of the IIQPQQPAQ sequence described by Arentz-Hansen et al. [18].

Of the nine Butte 86 full-length gamma gliadins, only one, encoded by Butte 86 contig #4, was a perfect match with a sequence in NCBI (Table 3). Four Butte 86 gamma gliadins (#2,3,6,7) had only one or two amino acid differences from sequences in NCBI while four (#1,5,8,10) had numerous amino acid changes. Proteins encoded by contigs assembled as part of TaGI Release 10.0, TaGI Release 11.0, US Wheat Genome Project, and HarvEST Version 1.14 were perfect matches with 5, 1, 1 or 6 of the full-length proteins encoded by Butte 86 contigs, respectively (Table 3). All of the assemblies and the NCBI non-redundant database also contained a perfect match to the incomplete protein encoded by Butte 86 contig #11.

**Table 3:** Comparison of gamma gliadins encoded by Butte 86 contigs to proteins in NCBI and proteins encoded by contigs from other EST assemblies.

Butte 86 Contig #	# Amino acids	NCBI Accession #^1^	Identity	TaGI 10.0 Contig #	Identity	TaGI 11.0 Contig #	Identity	US Wheat Genome Project Contig #	Identity^2^	HarvEST 1.14 Contig #	Identity^2^
1	328	[GenBank: AAK84774.1]	326/337	TC234710^3^	194/197	TC291857^3^	181/193	18524^4^	nd	14880	328/328
				TC250086	328/328	TC280138	327/328			1901^4,5^	nd
				TC250043	326/337						
2	327	[GenBank: AAK84779.1]	326/327	TC250310	327/327	TC327998	327/327	18871^4^	nd	4446	327/327
						TC333020^3^	160/198			14939^3,4^	nd
3	302	[GenBank: AAA34272.1]	300/302	TC249992	300/302	TC308951	299/302	18764	299/302	2079	302/302
4	302	[GenBank: ACJ03466.1]	302/302	TC250000	302/302	TC308951	276/302	18752	301/302	14939^3,4^	nd
						TC337354	269/302			14945^3^	212/230
5	358	[GenBank: ACJ03521.1]	342/362	TC233870	357/358	TC357319^6^	357/358	18873^7^	nd	15356	358/358
				TC250310	306/358						
6	285	[GenBank: ABO37962]	283/285	TC249991	285/285	TC329976^3^	254/264	18814	285/285	15101	285/285
						TC332920	265/285				
7	291	[GenBank: ACJ03428.1]	290/291	TC232926	291/291	TC296897	290/291	18753	289/291	4448	290/291
										1900^8^	291/291
8	332	[GenBank: AAK84774.1]	317/337	TC250043	317/337	TC280138	313/332	18524^4^	nd	14880	314/332
				TC234710^3^	194/197	TC281237	318/339	271^3^	176/176	4604^9^	nd
				TC250086	314/332	TC291857^3^	183/185			1901^4,5^	nd
										47432^3^	188/188
9^3^	289	[GenBank: ACJ03517.1]	266/294	TC249996^3^	204/227	TC293526	283/289	16926	288/289	14942	288/289
				TC250031^3^	251/255						
10	286	[GenBank: AAA34272.1]	284/302	TC249992	284/302	TC308951	283/302	18764	283/302	2079	286/302
						TC337760^3^	142/180	18842^4^	nd	209^5^	283/286
11^3^	163	[GenBank: ACJ03465.1]	163/163	TC233421	163/163	TC277980	163/163	16585^3^	163/163	4328	163/163

^1 ^accession with highest score in BLASTp search of non-redundant protein sequences using BLOSUM62, no compositional adjustments, no filters, no masks. Database last searched on 5/08/09.

^2 ^protein identities that were not determined because consensus sequences of contigs contained frameshifts or stop codons in the reading frame are indicated by nd.

^3 ^partial coding sequence.

^4 ^consensus sequence contains frameshift or stop codon in reading frame.

^5 ^complement of consensus sequence.

^6 ^contig contains a portion of a LMW-GS sequence at 5' end.

^7 ^encodes alpha gliadin.

^8 ^encodes identical protein to Butte 86 contig, but does not contain any ESTs from Butte 86.

^9 ^encodes LMW-glutenin subunit.

### Analysis of gamma gliadins containing an extra cysteine residue

**Figure 2:**
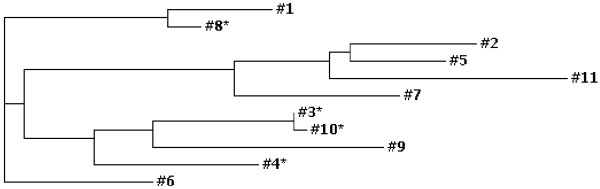
**Phylogram showing relationships among full-length gamma gliadins encoded by Butte 86 contigs**. Proteins containing nine cysteine residues are indicated with asterisks.

Of particular interest is the fact that proteins encoded by Butte 86 contigs #3, 4, 8 and 10 each contain an additional cysteine residue in the repetitive region of the protein 26 amino acids from the N-terminus. Seven of 18 ESTs that make up contig #3 and ten of 14 ESTs that make up contig #4 contain the TGC codon for the extra cysteine (Additional File 1). The TGC codon for the extra cysteine is found in only one EST in contig #8, [GenBank: BQ805093], and one EST in contig #10, [GenBank: BQ806189]. However, phred quality scores of 50, 42 and 42 for [GenBank: BQ805093] and 44, 42 and 42 for [GenBank: BQ806189] provide >99.99% probability that these bases were called correctly [19]. A phylogram of Butte 86 gamma gliadins (Figure 2) shows that proteins encoded by contigs #3, 4, and 10 are closely related to the protein encoded by contig #9 which does not contain the extra cysteine, while the gamma gliadin encoded by contig #8 is most similar to the protein encoded by contig #1 that does not contain the extra cysteine. The gamma gliadin encoded by Butte 86 contig #3 differs from that encoded by contig #4 by 28 amino acid substitutions that are spread throughout the protein, from the protein encoded by contig #10 by a 16 amino acid deletion in region II, and from the partial gamma gliadin encoded by contig #9 by a 5 amino acid deletion and 27 amino acid substitutions, one of which is a serine in place of a cysteine. The protein encoded by contig #8 differs from that encoded by contig #1 by 5 amino acid substitutions in the signal peptide, one substitution in region I, and 6 substitutions, one amino acid deletion and a 5 amino acid insertion that contains the additional cysteine residue in region II.

**Figure 3:**
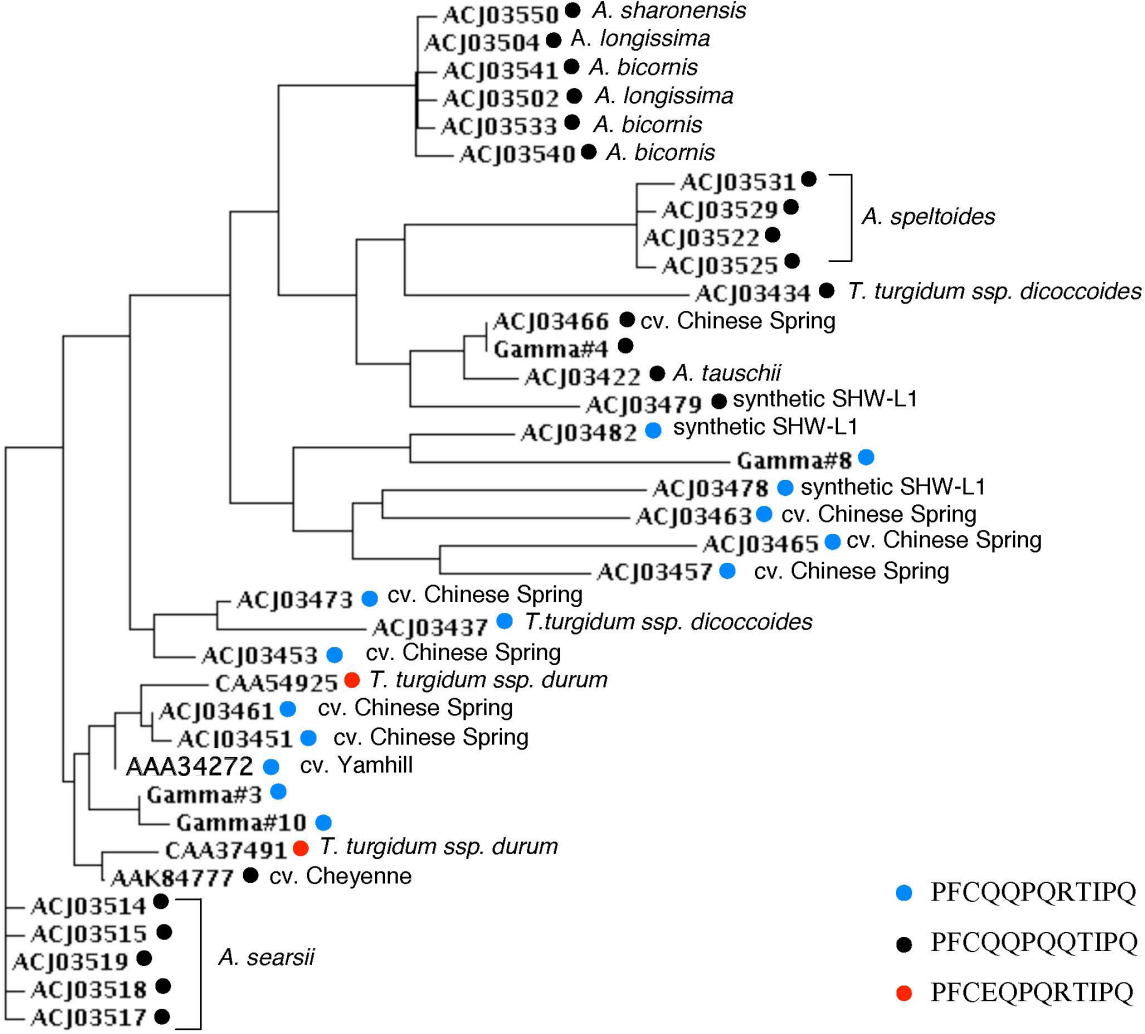
**Phylogram of gamma gliadins containing nine cysteine residues**. Proteins containing the sequence PFCQQPQRTIPQ are denoted with a blue circle, those containing PFCQQPQQTIPQ are denoted with a black circle and those containing PFCEQPQRTIPQ are denoted with a red circle. For proteins from diploid or tetraploid species, the source of the sequence is indicated in italics. For proteins from hexaploid wheat, only the cultivar is indicated.

In proteins encoded by Butte 86 contigs #3, 8 and 10, the extra cysteine residue is found in the peptide PFCQQPQRTIPQ while in the protein encoded by contig #4, the arginine at position 8 has been replaced by a glutamine (Figure 1). To determine the prevalence of gamma gliadins containing the additional cysteine in the NCBI non-redundant protein sequence database, a BLASTp search was performed of the database with the peptide PFCQQPQRTIPQ. This search revealed 33 gamma gliadin sequences that contain a cysteine residue in this region. Eleven sequences were identical in this region to proteins encoded by Butte 86 contigs #3, 8 and 10. These included seven genomic sequences from *T. aestivum *cv. Chinese Spring, two from the synthetic wheat SHW-L1, one from *T. aestivum *cv. Yamhill and one from *T. turgidum *ssp. *dicoccoides*. Twenty additional sequences matched PFCQQPQQTIPQ found in the protein encoded by Butte 86 contig #4. Most of these were from the diploid species, *Aegilops searsii *(5), *A. speltoides *(4), *A. bicornis*, (3), *A. longissima *(2), *A. sharonensis *(1) and *A. tauschii *(1), one was from the tetraploid *T. turgidum *ssp. *dicoccoides*, and one each was from the hexaploid *T. aestivum *cvs. Chinese Spring and Cheyenne and the synthetic wheat SHW-L1. In two additional sequences from *T. turgidum*, the extra cysteine was found within the peptide PFCEQPQRTIPQ. Thus far, gamma gliadins containing the extra cysteine have not been identified in *T. monococcum *or *T. urartu*, related species containing the A genome, although numerous gamma gliadin sequences from these species are included in NCBI. The 33 sequences, along with those encoded by Butte 86 contigs #3, 4, 8 and 10, were aligned using Clustal W and a phylogram was generated (Figure 3). Butte 86 gamma gliadins #3, 4 and 10 are most similar to proteins in other bread wheats while Butte 86 gamma gliadin #8 is most similar to a protein from a synthetic wheat.

In addition to the previously described sequences, ten other DNA sequences have been reported in NCBI that encode gamma gliadins containing an odd number of cysteine residues that could potentially be linked into the glutenin polymer. These include four genomic sequences that encode proteins that are missing one of the conserved cysteine residues in regions III, IV or V and six sequences that encode proteins that contain an additional cysteine residue at various locations in regions II or V (Table 4). To determine whether any of these gamma gliadins were likely to be expressed, a tBLASTn search was conducted of the 1,121,565 *Triticum *ESTs in NCBI using the amino acid sequence that surrounds the site of each added or deleted cysteine. A single EST was identified that corresponds to one of the gamma gliadins containing seven cysteines, [GenBank: CAC94868.1]. ESTs corresponding to two gamma gliadins containing nine cysteines [GenBank: ACI04102.1, ACI04105.1] also were found, suggesting that these may also be expressed. None of the ESTs were from Butte 86, suggesting that all of the gamma gliadins expressed in this cultivar that contain an odd number of cysteines have been identified. There is no evidence from current EST data that the other seven gamma gliadin variants are expressed.

**Table 4:** Evidence for expression of sequences encoding gamma gliadins with odd numbers of cysteine residues.

Protein accession #	Source	# Cysteine residues	Region of missing/additional cysteine	Reference	Sequence used to search NCBI^1^	# ESTs in NCBI ^2^
[GenBank: CAC94868.1]	*T. aestivum *cv. Mjoelner	7	III	Arentz-Hansen et al. [18]	DCQVMRQQSCQQLAQIP	1
[GenBank: ACJ03505.1]	*Aegilops longissima*	7	V	Qi et al. [20]	VLQTLPTMRNMYVPPY	0
[GenBank: ACJ03516.1]	*Aegilops searsii*	7	IV	Qi et al. [20]	LAQIPQQLQYAAIHS	0
[GenBank: ACI04088.1]	*T. aestivum *cv. Jinan 177	7	V	Chen et al. [21]	EAIRSLVLQTLPSMSNVYVPP	0
[GenBank: AAK84776.1]	*T. aestivum *cv. Cheyenne	9	II	Anderson et al. [14]	HTFPQPQQTCPHQPQQQFPQ	0
[GenBank: ACJ03499.1]	*T. urartu*	9	II	Qi et al. [20]	PFCQHQQPFYQQPQQTFPHQPQ	0
[GenBank: CAI78902.1]	*T. aestivum *cv. Neepawa	9	II	Snegaroff, unpublished	PFPQSQQQCLQQPQHQFPQPTQQ	0
[GenBank: ACI04102.1]	*T. aestivum *× *Lophopyrum elongatum*	9	V	Chen et al. [21]	RHTDPATTVSACPGQGIIQPQQ	3
[GenBank: ACI04105.1]	*T. aestivum *× *Lophopyrum elongatum*	9	V	Chen et al. [21]	RHTDPATTVSACPGQGIIQPQQ	3
[GenBank: ACI04108.1]	*T. aestivum *× *Lophopyrum elongatum*	9	II	Chen et al. [21]	PFPQPQQPFCQQPQQPYP	0

^1 ^The extra cysteine in proteins containing nine cysteines or residue substituted for cysteine in proteins containing seven cysteines is underlined.

^2 ^determined by tBLASTn search of non-human, non-mouse ESTs, limited to *Triticum*, word size 2, expect 30000, PAM30, no compositional adjustment, no filters, no masks, database last searched on 7/22/09.

### Identification of Butte 86 gamma gliadins by MS/MS

Because neither the NCBI database nor any single EST assembly contained sequences that matched all of the Butte 86 gamma gliadins, specialized databases that included the Butte 86 sequences were constructed for MS/MS identification of proteins from Butte 86 flour (described in Vensel et al., in preparation). In a pilot study, Butte 86 gamma gliadins #2, 4, 5, 6, 7 and 11 were distinguished with high levels of confidence by MS/MS in a 2% SDS extract from flour (Table 5). Overall peptide coverage ranged from 27.6% for gamma gliadin #11 to 56.5% for gamma gliadin #4 (Table 5, Additional File 3). Peptides identified by MS/MS that were not shared by other Butte 86 gamma gliadins were designated as unique peptides. For gamma gliadin #4, 13 of the 22 identified peptides were found only in this protein. Seven unique peptides were generated with chymotrypsin, five with thermolysin and one with trypsin. The additional cysteine residue that characterizes gamma gliadin #4 was found in one chymotryptic peptide indicated in italics in Table 5. For gamma gliadin #5, 31 peptides covered 44.8% of the protein sequence. Fifteen unique peptides were generated with chymotrypsin, three with thermolysin and four with trypsin. For gamma gliadin #7, 13 peptides accounted for 43.4% of the protein sequence. Of the unique peptides, five were chymotryptic fragments, while two each were generated with thermolysin and trypsin. Gamma gliadin #6 was distinguished by three unique chymotryptic peptides and eight thermolytic peptides. Gamma gliadin #2 was distinguished by four chymotryptic peptides and one thermolytic peptide and gamma gliadin #11 was distinguished on the basis of one chymotryptic peptide and one thermolytic peptide. Proteins in two bands could not be distinguished unequivocally from the MS/MS data. One band yielded four peptides generated with chymotrypsin, two with thermolysin and one with trypsin that are found only in Butte 86 gamma gliadins #3 and 10. Although both proteins contain the extra cysteine residue and overall coverage was more than 37%, peptides containing this cysteine were not observed. Another band yielded two peptides generated with chymotrypsin and one peptide generated with trypsin that are found only in Butte 86 gamma gliadins #1 and #8. However, in this case the data were not sufficient to distinguish Butte 86 gamma #1, containing eight cysteines, from gamma gliadin #8, containing nine cysteines.

**Table 5:** Butte 86 gamma gliadins identified by MS/MS from wheat flour.

Butte 86 contig #	Band # ^1^	Total # peptides ^2^	% Coverage^3^	Unique peptides^4^	Enzyme used to generate peptide^5^
2	126	14	36.0	SQQQQVGQGSL	CH
				QQLPQPQQPQQSFPQQQR	CH
				SQQQQVGQGSLVQGQGIIQPQQPAQL	CH
				NIQVDPSGQVQWLQQQLVPQLQQPL	CH
				LQQPQQPFPQPQQQLPQPQQPQQ	TH

4	188	22	56.5	SIIMQQEQRQGVQIRRPL	CH
				LQPQQPQQSFPQQQQPL	CH
				VSPDCSTINAPF	CH
				VSPDCSTINAPFASIVVGIGGQ	CH
				LQPQQPQQSFPQQQQPLIQL	CH
				LQPQQPQQSFPQQQQPLIQLSL	CH
				*CQQPQQTIPQPHQTF*	CH
				IIMQQEQRQG	TH
				IIMQQEQRQGVQ	TH
				LQPQQPQQSFPQQQQPLIQ	TH
				VDPGYQVHWPQQQPFPQPQQP	TH
				VHWPQQQPFPQPQQP	TH
				NFLLQQCNPVSLVSSLISMILPR	TR

5	145	31	44.8	VPPECSIIRAPF^6^	CH
				IQPSLQQR	CH
				IQPSLQQRL	CH
				SQQQQLGQGTL^6^	CH
				QSFPQQQRPF	CH
				ILLPLSQQQQL^6^	CH
				TQQPQQPFPQFQQPHQPF^6^	CH
				VQGQGIIQPQQLAQLEAIRSL^6^	CH
				SQQQQLGQGTLVQGQGIIQPQQL^6^	CH
				SQQQQLGQGTLVQGQGIIQPQQLAQLEAIRSL^6^	CH
				ILLPLSQQQQLGQGTL^6^	CH
				SQQQQLGQGTLVQGQGIIQPQQLAQL^6^	CH
				SQQPQQAFPQPQQTFPHQPQQQVPQPQQPQQPF	CH
				HQPQQQFPQPQQPQQSFPQQQRPF	CH
				HQPQQQFPQPQQPQQSFPQ	CH
				AFPQPQQTFPHQPQQQVPQPQQPQQPF	TH
				FHQPQQQFPQPQQPQQ	TH
				FHQPQQQFPQPQQPQQSFPQQQRP	TH
				IQPSLQQR	TR
				QLAQLEAIR	TR
				PFIQPSLQQR	TR
				QSFPQQQRPFIQPSLQQR	TR

6	186	16	36.5	RQPQQPF	CH
				ASIVAGISGQ	CH
				YQQPQQTFPQPQ	CH
				LAQIPRQ	TH
				IQILRPLFQ	TH
				IIQPQQPAQYEVIRS	TH
				FRQPQQPFY	TH
				IIQPQQPAQYE	TH
				YQQPQQTFPQPQQ	TH
				FRQPQQPFYQQPQQTFPQPQQ	TH
				FYQQPQQTFPQPQQ	TH

7	170	13	43.4	LQPHQPF	CH
				ASIVASIGGQ	CH
				QGVQILVPL	CH
				SQQQQVGQGIL	CH
				SQQQQVGQGILVQGQGIIQPQQPAQL	CH
				VYVPPYCST	TH
				LQPHQPFSQQPQQ	TH
				APFASIVASIGGQ	TR
				QPFPQQPQQPYPQQPQQPFPQTQQPQQPFPQSK	TR

11	149	4	27.6	YQQQQVGQGTLVQGQGIIQPQQPAQL	CH
				SFPQQQPPF	TH

1^7^	180	7	24.9	ASIVADIGGQ	CH
8^7^			22.0	KAPFASIVADIGGQ	CH
				APFASIVADIGGQ	TR

3^8^	169	16	37.5	ANIDAGIGGQ	CH
10^8^			39.7	GIIQPQQPAQLEGIRSLVL	CH
				VPPNCSTINVPY	CH
				VPPNCSTINVPYANIDAGIGGQ^6^	CH
				VTILRPLFQ^6^	TH
				INVPYANIDAGIGGQ	TH
				NFLLQQCNHVSLVSSLVSIILPR	TR

^1 ^detailed in Vensel et al. (in preparation).

^2 ^obtained by interrogation of "subset" database with MS/MS data.

^3 ^excluding 19 amino acid signal peptide.

^4 ^sequences of peptides obtained by MS/MS that are not shared by other Butte 86 gamma gliadins. Peptide containing the extra cysteine is indicated in italics.

^5 ^CH, chymotrypsin; TH, thermolysin; TR, trypsin.

^6 ^peptides that distinguish Butte 86 sequence from closest NCBI match.

^7 ^MS/MS data consistent with either Butte 86 gamma gliadin #1 or #8.

^8 ^MS/MS data consistent with either Butte 86 gamma gliadin #3 or #10.

## Discussion

The complement of gamma gliadin genes expressed in a single US bread wheat cultivar was examined by assembling nine complete and two partial coding regions from Butte 86 ESTs identified as gamma gliadin sequences in TaGI Release 10.0. Analysis of the encoded proteins highlighted the variability among gamma gliadins expressed within a single cultivar. The study also identified the sequences of four Butte 86 gamma gliadins that contain an additional cysteine residue in region II in addition to the eight conserved cysteines in regions III and V. More importantly, this work facilitated the discrimination of closely related proteins in Butte 86 flour by MS/MS, including gamma gliadins containing the additional cysteine residue.

The NCBI database contains the sequences of more than 300 gamma gliadins from *T. aestivum *and related species (last searched 6/10/09). Yet, the data indicate that these represent only a portion of the sequence heterogeneity present in this group of gluten proteins. Only one of the nine Butte 86 contigs encoded a complete protein that was a perfect match with a sequence in NCBI. Moreover, the best match in NCBI to one of the most highly expressed gamma gliadins in Butte 86 (#5) was to a recently reported sequence from *Aegilops speltoides *[GenBank: ACJ03521] that differs by 20 amino acids [20]. This may be explained in part by the fact that many recent gamma gliadin sequences were obtained by amplification of either genomic DNA [20,21] or cDNA [22] and thus reflect variants of sequences already in the database. In addition, there are only 20 cDNA sequences that encode complete gamma gliadin proteins in NCBI. These include nine cDNAs amplified from different *T. aestivum *and *T. turgidum *lines by Piston et al. [22], three cDNAs from *T. aestivum *cv. Cheyenne [14], one from *T. aestivum *cv. Chinese Spring [23], six from *T. monococcum *[24] and one from *T. turgidum *subsp. durum × *Hordeum chilense*.

Because more than 3,600 ESTs were generated from Butte 86 developing grain as part of a large scale sequencing effort [25], it was possible to evaluate the complement of gamma gliadins expressed in this cultivar by analysis of publicly available EST data. In addition to eliminating the inherent bias that results from amplification of sequences by PCR, the analysis provides new information regarding the expression levels of the genes. The data demonstrate that there is differential expression of the many closely related members of the gamma gliadin gene family during grain development. Based on the number of ESTs that comprise each of the contigs, the two most highly expressed gamma gliadins in Butte 86 were #2 and #5. It is interesting that the proteins encoded by contigs #2 and 5 cluster with the group II gamma gliadins described by Piston et al. [22] (data not shown) that were found to be expressed at much higher levels than other groups of gliadins in the bread wheat cvs. Anza and Perico. The data also suggests that gamma gliadins that can be incorporated into the glutenin polymer are quite prevalent in Butte 86. Four of the contigs from Butte 86 encoded gamma gliadins that contained nine cysteines and at least two of these were expressed at moderate levels. This finding suggests that the ratio of different types of gamma gliadins could play a significant role in flour quality. It will be particularly interesting to determine whether gene expression correlates with protein accumulation in Butte 86 grain. Additionally, there is a need to determine the ratios of the two types of gamma gliadins in other cultivars. Given the wealth of information contained in current EST databases, careful analyses of ESTs from single cultivars could provide a better representation of expressed gamma gliadin genes as well as new information about their levels of expression.

The Butte 86 ESTs are contained in several large assemblies that include sequences from many different cultivars and related wheat species. The analysis revealed that no single assembly included contigs encoding gamma gliadins that match perfectly with those encoded by all of the Butte 86 contigs. This is not surprising given that the collections of ESTs and the assembly methods of the databases differ. Additionally, gamma gliadins present unique challenges for EST assembly because of the repetitiveness of their sequences. It is interesting that the assignment of individual ESTs to contigs by the US Wheat Genome Project assembly was most consistent with the Butte 86 assignments. However, consensus sequences frequently included stop codons or frameshifts in reading frames and only one full-length protein and one partial protein encoded by consensus sequences from this assembly were perfect matches with Butte 86 sequences (Table 3). While the HarvEST 1.14 assembly included many of the same sequences as the US Wheat Genome Project assembly, ESTs from six Butte 86 gamma gliadin contigs were assigned to single contigs in this assembly and six of the full-length proteins and one partial protein encoded by the HarvEST 1.14 consensus sequences were identical to Butte 86 gamma gliadins. Releases 10.0 and 11.0 of TaGI include a much larger collection of ESTs. Assignment of ESTs to contigs in TaGI Release 10.0 was more consistent with the Butte 86 assignments than in TaGI Release 11.0, and more of the proteins encoded by the consensus sequences were identical to Butte 86 full-length gamma gliadins. Clearly, the complexity of the gliadin sequences demands that special attention be paid to the assembly of ESTs into contigs.

For proteomics studies aimed at distinguishing closely related gluten proteins, it is essential that the sequences of the complement of proteins expressed in a given cultivar are included in the database used for analysis of MS/MS data, so that spectral data will yield a maximum number of peptide identifications with high probabilities and cover a significant portion of the protein sequence. The specialized database constructed for analysis of MS/MS data contained more than 2.5 million protein sequences, some of which were redundant. It is reassuring that six gamma gliadin proteins identified from flour by MS/MS with high levels of confidence correspond to Butte 86 sequences. In our pilot study, 31 peptides with probabilities greater than 90% accounted for 44.8% of Butte 86 gamma gliadin #5. Nine of these peptides differ between gamma gliadin #5 and its closest match in NCBI, [GenBank: ACJ03521.1] (Table 5). Had the Butte 86 sequences not been included in the database used for interrogation of MS/MS data, some spectra would not have yielded valid peptide identifications and sequence coverage of the protein would have been less than 20%. One of the moderately expressed gamma gliadins in Butte 86 (#3) differs from a protein in *T. aestivum *cv. Yamhill [GenBank: AAA34272.1] by only 2 amino acids. Three of the 16 peptides identified by MS/MS included these amino acids. Thus, inclusion of the Butte 86 sequences in the database used for analysis of MS/MS data made it possible to distinguish proteins from the two cultivars.

Distinguishing gamma gliadins that are likely to be monomeric proteins from those that contain an odd number of cysteine residues and are able to participate in the formation of glutenin polymers is far from trivial. Muccilli et al. [26] used MALDI-TOF to characterize proteins in LMW-GS fractions from the bread wheat cv. Chinese Spring and found a number of proteins with MWs in the gamma gliadin range that had nine cysteine residues. Ferrante et al. [27] verified that a gene encoding a gamma gliadin with nine cysteines was expressed in durum wheat by first expressing the gene in *E. coli*. A protein from flour with similar mobility in 2-DE to the *E. coli *protein was then selected, analyzed by MS/MS and showed good agreement with the protein encoded by the gene. This approach was successful in part because both the gene sequence and the identified protein were from the same durum cultivar. Gobaa et al. [28] concluded that a protein over-expressed in double haploid lines containing a 1BL.1RS translocation was a gamma gliadin containing nine cysteines. The identification of the protein was based on the best match of spectra from seven peptides to Swiss-Prot and NCBI non-redundant databases. However, none of the identified peptides contained the additional cysteine and only two of the reported peptides were perfect matches with [Swiss-Prot: P21292], the gamma gliadin containing nine cysteines. For many proteins, a valid MS/MS identification can be obtained if at least two non-redundant peptides with 95% probability scores match a single entry in the database that is searched. However, in the case of gamma gliadins where many proteins differ by only a few amino acids, two peptides may not be sufficient to associate a protein with a specific gene sequence unless the peptides are from polymorphic regions. It is interesting that the two peptides from the Gobaa et al. [28] study that matched [Swiss-Prot: P21292], QQQMN and TLPTMCNVYV, are found in seven gamma gliadins from Butte 86, some of which contain eight cysteines and some of which contain nine cysteines. Thus, it is difficult to say that the protein was a nine cysteine gamma gliadin without having identified a peptide containing the extra cysteine or having compared the spectral data to gamma gliadin sequences from the cultivar under study.

Methods for MS/MS data analysis evolve rapidly. Search algorithms that match the MS/MS spectra of peptides with protein sequence information from databases are constantly being improved. The use of multiple search engines also can increase the number of peptide identifications and the validity of protein identifications [29]. The choice of database to be searched is critical, particularly in organisms where a completely annotated genome sequence is not available. In addition, new sequences are constantly being added to gene and protein databases. Indeed, since the work reported in this manuscript was initiated, several hundred new gamma gliadin sequences have been reported in the NCBI non-redundant database [20,21,24] and the TaGI assembly was updated to Version 11.0 that included over 400,000 new sequences. In the end, careful interpretation of MS/MS data is essential for evaluating its biological significance. For protein groups as complex as the gamma gliadins, there is no question that detailed sequence information from the cultivar under study facilitates these efforts.

## Conclusions

2-DE and other analytical techniques can be used to separate and quantify gamma gliadins of interest in a particular study [5,6,30]. However, to be able to put the results from these studies into a biological context, it is necessary to distinguish very closely related proteins, some of which differ in the number of cysteine residues that are available for disulfide bond formation. The considerable heterogeneity observed within the gamma gliadin protein group suggests that it is important to have information about the complement of gamma gliadin genes expressed in the cultivar under study. Undoubtedly, the identification of marker peptides for single gamma gliadin gene products from Butte 86 will prove valuable in future quantitative studies aimed at comparing the levels of specific gamma gliadins in grain from plants grown under different environmental conditions.

## Methods

ESTs representing gamma gliadins in *Triticum aestivum *cv. Butte 86 were identified by searching the Dana Farber Cancer Institute *Triticum aestivum *Gene Index (TaGI) Release 10.0 [8] with the keywords "gamma gliadin". Of the fifty-nine gamma gliadin contigs returned in the search, seventeen included ESTs from Butte 86. A total of 153 Butte 86 ESTs were identified. These ESTs were aligned using GeneWorks 2.5 (Oxford Molecular Group, Campbell, CA) and new gamma gliadin contigs were formed. Default settings were cost to open gap = 2, cost to lengthen gap = 4, minimum diagonal length = 4 and maximum diagonal offset = 190. Assemblies were inspected manually and consensus sequences were generated after resolving mismatches that occurred in overlap regions of ESTs. All mismatches were resolved by examining phred quality scores for individual ESTs obtained by searching the file 'TA059E1X.fasta.qual' downloaded from [31] with the original clone name for each EST. Quality scores >30, indicating a 99.9% accuracy of the base call, were considered valid [19]. Each consensus sequence was examined for open reading frames and translated using functions in GeneWorks 2.5. Sequence alignments were performed using ClustalW2 with default settings [32]. DNA and protein homology searches were performed using BLASTn, tBLASTn and BLASTp from NCBI [33].

Extraction and fractionation of flour proteins from *T. aestivum *cv. Butte 86 and MS/MS analyses of proteins are described in detail in Vensel et al. (in preparation). In short, a 2% SDS protein fraction was prepared from Butte 86 flour and separated by RP-HPLC as described by Dupont et al. [34]. Proteins in individual peaks were collected and analyzed by SDS-PAGE. Individual bands were excised from gels, digested with chymotrypsin, thermolysin or trypsin and introduced by nanoflow RP-HPLC ESI into a QSTAR Pulsar *i *mass spectrometer (Applied Biosystems, Foster City, CA). MS/MS spectra generated from each digest were used to interrogate a "SuperWheat" database. The "SuperWheat" database contained NCBI non-redundant green plant protein sequences, translated sequences (in all six reading frames) of contigs from TaGI Releases 10.0 and 11.0, US Wheat Genome Project, HarvEST 1.14 (WI all NSF "stringent" assembly from 05/08/04), NCBI Unigene Build #55, and all ESTs from Butte 86 developing grain, as well as translated sequences (reading frame only) of a selection of Butte 86 contigs, including those assembled in this study. The total number of protein sequences in the "SuperWheat" database was 2,562,722. Two search engines, X!Tandem [35] and Mascot Daemon [36], were used for analysis of spectral data. Scaffold Version 2_02_04 [37] was used to compile MS/MS based peptide and protein identifications. The sequences of all proteins that matched spectra in the dataset were then exported from Scaffold and used to construct a smaller "subset" database. These 1,526 sequences were combined with the set of translated sequences from Butte 86 contigs and an equal number of decoy protein sequences from the bacterium *Methanosarcina mazei*, and the resulting database was interrogated with the original spectral data using the same search engines. Peptide identifications were accepted if they could be established at greater than 90.0% probability as specified by the Peptide Prophet algorithm [38] with a parent mass tolerance threshold of 100 ppm. Protein identifications were considered further if they could be established at greater than 95.0% probability and contained at least 2 identified peptides. Protein probabilities were assigned by the Protein Prophet algorithm [39]. Because of the complexity of the gamma gliadin protein families and sequence redundancy within the databases, peptide data were further inspected manually. For each protein band, individual gamma gliadin peptides obtained from interrogation of the 'subset' database were used to search against the sequences of all Butte 86 gamma gliadins. Peptides found within a protein encoded by only one Butte 86 gamma gliadin contig were considered unique. Peptides found in more than one Butte 86 gamma gliadin sequence were assigned to proteins distinguished by unique peptides in the band or to the least number of proteins that accounted for all peptides.

## Abbreviations

2-DE: 2-dimensional gel electrophoresis; bp: base pair; ESI: electrospray ionization; EST: expressed sequence tag; HMW: high molecular weight; LMW: low molecular weight; MALDI-TOF: matrix-assisted laser desorption/ionization time of flight mass spectrometry; MS/MS: tandem mass spectrometry; MW: molecular weight; NCBI: National Center for Biotechnology Information; PCR: polymerase chain reaction; RP-HPLC: reverse phase-high pressure liquid chromatography; SDS-PAGE: SDS polyacrylamide gel electrophoresis; TaGI: *Triticum aestivum *Gene Index.

## Authors' contributions

SBA performed all sequence analyses and comparisons and drafted the manuscript. WHV conducted MS/MS experiments and assembled the "SuperWheat" and "subset" databases. FMD fractionated wheat flour proteins used in MS/MS analyses and contributed to experimental design. All authors analyzed MS/MS data and have read and approved the final manuscript.

## Supplementary Material

Additional file 1 Assignment of Butte 86 ESTs to contigs reported in this paper and to contigs from other EST assemblies. Click here for file

Additional file 2 Consensus sequences of Butte 86 contigs encoding gamma gliadins. Click here for file

Additional file 3 **Amino acid sequences of gamma gliadins deduced from consensus sequences of Butte 86 contigs. **Peptides identified by MS/MS after digestion with chymotrypsin are indicated in bold, thermolysin are underlined, and trypsin are enclosed in boxes. Click here for file

## References

[B1] Shewry PR Tatham AS The prolamin storage proteins of cereal seeds: structure and evolution Biochem J 1990 267 1 12 2183790 10.1042/bj2670001 PMC1131235

[B2] Wieser H Chemistry of gluten proteins Food Microbiol 2007 24 115 119 10.1016/j.fm.2006.07.004 17008153

[B3] D'Ovidio R Masci S The low-molecular-weight glutenin subunits of wheat gluten J Cereal Sci 2004 39 321 339 10.1016/j.jcs.2003.12.002

[B4] Kasarda DD Pomeranz Y Glutenin structure in relation to wheat quality Wheat is unique 1989 St. Paul, Minn.: American Association of Cereal Chemists 277 302

[B5] DuPont FM Hurkman WJ Vensel WH Chan R Lopez R Tanaka CK Altenbach SB Differential accumulation of sulfur-rich and sulfur-poor wheat flour proteins is affected by temperature and mineral nutrition during grain development J Cereal Sci 2006 44 101 112 10.1016/j.jcs.2006.04.003

[B6] Dupont FM Hurkman WJ Vensel WH Tanaka C Kothari KM Chung OK Altenbach SB Protein accumulation and composition in wheat grains: effects of mineral nutrients and high temperature Eur J Agron 2006 25 96 107 10.1016/j.eja.2006.04.003

[B7] Ferranti P Mamone G Picariello G Addeo F Mass spectrometry analysis of gliadins in celiac disease J Mass Spectrom 2007 42 1531 1548 10.1002/jms.1361 18085572

[B8] DFCI Wheat Gene Index http://compbio.dfci.harvard.edu/tgi/cgi-bin/tgi/gimain.pl?gudb=wheat

[B9] Chao S Lazo GR You F Crossman CC Hummel DD Lui N Laudencia-Chingcuanco D Anderson JA Close TJ Dubcovsky J Gill BS Gill KS Gustafson JP Kianian SF Lapitan NLV Nguyen HT Sorrrells ME McGuire PE Qualset CO Anderson OD Use of a large-scale *Triticeae *expressed sequence tag resource to reveal gene expression profiles in hexaploid wheat (*Triticum aestivum *L.) Genome 2006 49 531 544 10.1139/G06-003 16767178

[B10] GrainGenes-SQL Query resources: contig information http://wheat.pw.usda.gov/cgi-bin/westsql/contig.cgi

[B11] HarvEST http://harvest.ucr.edu/

[B12] Huang X Madan A CAP3: a DNA sequence assembly program Genome Res 1999 9 868 877 10.1101/gr.9.9.868 10508846 PMC310812

[B13] SignalP 3.0 Server http://www.cbs.dtu.dk/services/SignalP/

[B14] Anderson OD Hsia CC Torres V The wheat γ-gliadin genes: characterization of ten new sequences and further understanding of γ-gliadin gene family structure Theor Appl Genet 2001 103 323 330 10.1007/s00122-001-0551-3

[B15] Qiao S-W Bergseng E Molberg O Jung G Fleckenstein B Sollid LM Refining the rules of gliadins T cell epitope binding to the disease-associated DQ2 molecule in celiac disease: Importance of proline spacing and glutamine deamidation J Immunol 2005 175 254 261 15972656 10.4049/jimmunol.175.1.254

[B16] Molberg O Uhlen AK Jensen T Solheim Flaete N Fleckenstein B Arentz-Hansen H Raki M Lundin KEA Sollid LM Mapping of gluten T cell epitopes in the bread wheat ancestors: implications for celiac disease Gastroenterology 2005 128 393 401 10.1053/j.gastro.2004.11.003 15685550

[B17] Sjostrom H Lundin KE Molberg O Korner R McAdam SN Anthonsen D Quarsten H Noren O Roepstorff P Thorsby E Sollid LM Identification of a gliadin T cell epitope in coeliac disease: general importance of gliadin deamidation for intestinal T cell recognition Scand J Immunol 1998 48 111 115 10.1046/j.1365-3083.1998.00397.x 9716100

[B18] Arentz-Hansen H McAdam SN Molberg O Fleckenstein B Lundin KEA Jorgensen TJD Jung G Roepstorff P Sollid LM Celiac lesion T cells recognize epitopes that cluster in regions of gliadins rich in proline residues Gastroenterology 2002 123 803 809 10.1053/gast.2002.35381 12198706

[B19] Ewing B Green P Base-calling of automated sequencer traces using *Phred*. II Error probabilities Genome Res 1998 8 186 194 9521922

[B20] Qi P-F Wei Y-M Ouellet T Chen Q Tan X Zheng Y-L The gamma-gliadin multigene family in common wheat (*Triticum aestivum*) and its closely related species BMC Genomics 2009 10 168 10.1186/1471-2164-10-168 19383144 PMC2685405

[B21] Chen F Zhao F Liu S Xia G The γ-gliadin gene content of a derivative from a somatic hybrid between bread wheat and tall wheatgrass Mol Breed 2009 24 2 117 126 10.1007/s11032-009-9275-x

[B22] Piston F Dorado G Martin A Barro F Cloning of nine γ-gliadin mRNAs (cDNAs) from wheat and the molecular characterization of comparative transcript levels of γ-gliadin subclasses J Cereal Sci 2006 43 120 128 10.1016/j.jcs.2005.07.002

[B23] Bartels D Altosaar I Harberd NP Barker RF Thompson RD Molecular analysis of γ-gliadin gene families at the complex *Gli-1 *locus of bread wheat (*T. aestivum *L.) Theor Appl Genet 1986 72 845 853 10.1007/BF00266556 24248211

[B24] Vaccino P Becker H-A Brandolini A Salamini F Kilian B A catalogue of *Triticum monococcum *genes encoding toxic and immunogenic peptides for celiac disease patients Mol Genet Genomics 2009 281 289 300 10.1007/s00438-008-0412-8 19104838 PMC2757618

[B25] Zhang D Choi DW Wanamaker S Fenton RD Chin A Malatrasi M Turuspekov Y Walia H Akhunov E Kianian P Otto C Simons K Deal K Echenique V Stamova B Ross K Butler E Doherty L Verhey S Johnson R Altenbach S Kothari K Tanaka C Shah MM Laudencia-Chingcuanco D Gitt M Pham J Han P Miller RE Crossman CC Chao S Lazo GR Klueva N Gustafson JP Kianian SF Dubcovsky J Walker-Simmons MK Gill KS Dvorak J Anderson OD McGuire P Qualset CO Nguyen HT Close TJ Construction and evaluation of cDNA libraries for large scale EST sequencing in wheat (*Triticum aestivum *L.) Genetics 2004 168 595 608 10.1534/genetics.104.034785 15514038 PMC1448820

[B26] Muccilli V Cunsolo V Saletti R Foti S Masci S Lafiandra D Characterization of B- and C-type low molecular weight glutenin subunits by electrospray ionization mass spectrometry and matrix-assisted laser desorption/ionization mass spectrometry Proteomics 2005 5 719 728 10.1002/pmic.200401029 15682464

[B27] Ferrante P Masci S D'Ovidio R Lafiandra D Volpi C Mattei B A proteomic approach to verify in vivo expression of a novel γ-gliadin containing an extra cysteine residue Proteomics 2006 6 1908 1914 10.1002/pmic.200500236 16485260

[B28] Gobaa S Bancel E Kleijer G Stamp P Branlard G Effect of the 1BL.1RS translocation on the wheat endosperm as revealed by proteomic analysis Proteomics 2007 7 4349 4357 10.1002/pmic.200700488 17973293

[B29] Searle BC Turner M Nesvizhskii AI Improving sensitivity by probabilistically combining results from multiple MS/MS search methodologies J Proteome Res 2008 7 245 253 10.1021/pr070540w 18173222

[B30] Mamone G Addeo F Chianese L Di Luccia A De Martino A Nappo A Formisano A De Vivo P Ferranti P Characterization of wheat gliadin proteins by combined two-dimensional gel electrophoresis and tandem mass spectrometry Proteomics 2005 5 2859 2865 10.1002/pmic.200401168 15952231

[B31] Index of/NSF/curator/quality/TA059E1X http://wheat.pw.usda.gov/NSF/curator/quality/TA059E1X/

[B32] ClustalW2 http://www.ebi.ac.uk/Tools/clustalw2/index.html?

[B33] Basic Local Alignment Search Tool http://blast.ncbi.nlm.nih.gov/Blast.cgi

[B34] DuPont FM Samoil V Chan R Extraction of up to 95% of wheat (Triticum aestivum) flour protein using warm sodium dodecyl sulfate (SDS) without reduction or sonication J Agric Food Chem 2008 56 7431 7438 10.1021/jf800776b 18616274

[B35] The Global Proteome Machine Organization Proteomics Database and Open Source Software http://www.thegpm.org/

[B36] Mascot Daemon http://www.matrixscience.com/daemon.html

[B37] Proteome Software Scaffold 2 http://www.proteomesoftware.com/Proteome_software_prod_Scaffold.html

[B38] Keller A Nesvizhskii AI Kolker E Aebersold R Empirical statistical model to estimate the accuracy of peptide identifications made by MS/MS and database search Anal Chem 2002 74 5383 5392 10.1021/ac025747h 12403597

[B39] Nesvizhskii AI Keller A Kolker E Aebersold R A statistical model for identifying proteins by tandem mass spectrometry Anal Chem 2003 75 4646 4658 10.1021/ac0341261 14632076

