# Supplementary material for: Analysis of expressed sequence tags from a single wheat cultivar facilitates interpretation of tandem mass spectrometry data and discrimination of gamma gliadin proteins that may play different functional roles in flour
Source: BMC Plant Biol. 2010 Jan 11;10:7. doi: 10.1186/1471-2229-10-7 (PMC2827424; doi:10.1186/1471-2229-10-7)
Supplement: Additional file 2 — Consensus sequences of Butte 86 contigs encoding gamma gliadins. [file 1471-2229-10-7-S2.DOC]

Additional File 2.

>Butte_Gamma#1

ACTCGAGCACAATCATCAAATCCAAGTAAGTAATAGTTAACATAAATCCACCATGAAGACCTTACTCATCCTGACAATCATTGCGGTGGCACTAACTACCACCACCGCCAATATACAGGTCGACCCTAGTGGCCAAGTACAATGGCCACAACAACAACAACCATTCCCCCAGCCCCAACAACCACAACAAATTTTTCCCCAACCCCAACAAACATTCCCCCATCAACCACAACAAGCATTTCCTCAACCCCAACAAACATTCCCCCATCAACCACAACAACAATTTCCCCAGCCCCAGCAACCACAACAACCATTTCCCCAGCAACCACAACAACAATTTCCCCAGCCCCAACAACCACAACAACCATTTCCCCAGCAACCACAACAACAATTTCCCCAGCCCCAACAACCACAACAACCATTTCCCCAGCCCCAACAACCCCAACTACCATTTCCGCAACAACCACAACAACCATTCCCCCAGCCTCAACAACCCCAACAACCATTTCCCCAGTTACAGCAACCACAACAACCTTTACCCCAGCCCCAACAACCGCAACAACCATTCCCCCAGCAACAACAACCATTGATTCAGCCATACCTACAACAACAGATGAACCCCTGCAAGAATTACCTCTTGCAACAATGCAACCCTGTGTCATTGGTGTCATCCCTCGTGTCAATGATCTTGCCACGAAGTGATTGCAAGGTGATGCGGCAACAATGTTGCCAACAACTAGCACAGATTCCTCAGCAGCTCCAGTGCGCAGCCATCCATGGCGTCGTGCATTCCATCATCATGCAGCAAGAACAACAACAACAACAACAACAACAACAAGGCATACAGATCATGCGGCCACTATTTCAGCTCGTCCAGGGTCAGGGCATCATCCAACCTCAACAACCAGCTCAATTGGAGGTGATCAGGTCATTGGTATTGGGAACTCTTCCAACCATGTGCAACGTGTTTGTTCCACCTGAGTGCTCCACCACCAAGGCACCATTTGCCAGCATAGTCGCCGACATTGGTGGCCAATGAAAAATGCAAGCGTTATGCTAATAGGTAGATGGATCATCGTTGCTTAGTTGATGCACCAATCGTTGTAACGATGAAAAATAAAGTGGTGTGCACCATCATGTGTGACCCCGACCAGTGCTAGTTCAAGCTTGGGAATAAAAGACAAACAAAGTTCTTGTTTGCTAGCAAAAAAAAAAAAAAA

>Butte_Gamma#2

ATTCGGACGAGGCACACTAGAGCACAAGCAGAAAATCAAAGTACGTAGTAGTTAACGCAAATCCACCATGAAGACCTTACTCATCCTGACAATCCTTGCGATGGCAATAACCATCGGCACCGCCAATATCCAGGTCGACCCTAGCGGCCAAGTACAATGGCTACAACAACAACTAGTCCCCCAGCTCCAACAGCCATTATCCCAGCAACCACAACAAACATTTCCCCAACCTCAACAAACATTCCCCCATCAACCACAACAACAAGTTCCCCAGCCTCAGCAACCACAACAACCATTTCTCCAGCCCCAACAACCATTCCCCCAACAACCACAACAACCATTCCCCCAGACTCAACAACCACAACAACCATTTCCCCAGCAACCACAACAACCATTTCCCCAGACTCAACAACCCCAACAACCATTTCCCCAACAACCACAACAACCATTCCCCCAGACTCAACAACCCCAACAACCATTTCCCCAGCTCCAGCAACCACAACAACCTTTTCCCCAGCCCCAACAACAATTACCGCAGCCCCAACAACCGCAACAATCATTCCCCCAACAACAACGGTCATTCATTCAACCATCTCTACAACAACAGTTGAACCCATGCAAGAATATCCTCTTGCAACAATGCAAACCTGCGTCATTGGTGTCATCCCTCTGGTCAATAATCTGGCCACAAAGCGATTGCCAAGTGATGCGGCAACAATGCTGCCAACAACTAGCACAGATTCCTCAACAGCTCCAGTGCGCAGCCATCCATAGCGTCGTGCATTCCATCATCATGCAGCAGCAGCAACAACAACAACAACAACAAGGCATGCATATCTTTCTGCCACTATCTCAGCAGCAACAGGTGGGTCAAGGTTCTCTAGTCCAAGGCCAGGGCATCATCCAACCACAACAACCAGCTCAATTGGAGGCGATCAGATCATTGGTGTTGCAAACTCTTCCATCCATGTGCAACGTGTATGTCCCACCAGAGTGCTCCATCATGAGGGCACCATTTGCCAGCATAGTCGCGGGCATTGGTGGCCAATGAAAAACTGAAGAGCTATACTAATAGGTAGATGGATCATCGTTGCTTAGCTGATGCACCAATCGATGTAACGATGACAAATAAAGTGGAGTGCACCATCATGTGTGACACCGGCCAGTGCTAGTTCAAGCTTGGGAATAAAAGACAAACAAAGTTCTTGTTTGCCAAAAAAAAAAAAAAAAAAAAAAAAA

>Butte_Gamma#3

CAAGCATCAAATCCAAGTAAGTATTAGTTAACGCAAATCCACCATGAAGACCTTACTCATCCTAACAATCCTTGCGATGGCAACAACCATCGCCACCGCCAATATGCAAGTCGACCCCAGCGGCCAAGTACAATGGCCACAACAACAACCATTCCCCCAGCCCCAACAACCATTCTGCCAGCAACCACAACGAACTATTCCCCAACCCCATCAAACATTCCACCATCAACCACAACAAACATTTCCCCAACCCCAACAAACATACCCCCATCAACCACAACAACAATTTCCCCAGACCCAACAACCACAACAACCATTTCCCCAGCCCCAACAAACATTCCCCCAACAACCCCAACTACCATTTCCCCAACAACCCCAACAACCATTCCCCCAGCCTCAGCAACCCCAACAACCATTTCCCCAGTCACAACAACCACAACAACCTTTTCCCCAGCCCCAACAACAATTTCCGCAGCCCCAACAACCACAACAATCATTCCCCCAACAACAACAACCGGCGATTCAGTCATTTCTACAACAACAGATGAACCCCTGCAAGAATTTCCTCTTGCAGCAATGCAACCATGTGTCATTGGTGTCATCTCTCGTGTCAATAATTTTGCCACGAAGTGATTGCCAGGTGATGCAGCAACAATGTTGCCAACAACTAGCACAAATTCCTCAACAGCTCCAGTGCGCAGCCATCCACAGCGTCGCGCATTCCATCATCATGCAACAAGAACAACAACAAGGCGTGACGATCCTGCGGCCACTATTTCAGCTCGCCCAGGGTCTGGGTATCATCCAACCTCAACAACCAGCTCAATTGGAGGGGATCAGGTCATTGGTATTGAAAACTCTTCCAACCATGTGCAACGTGTATGTGCCACCTAACTGCTCCACCATCAACGTACCATATGCCAACATAGACGCTGGCATTGGTGGCCAATGAAAAATGCAAGATCATCGTTGCTTAGCTGATGCACCAATCGTTGTAGCGATGACAAATAAAGTGTGCACCATCATGTGTGACCCCGACCAGTGCTAGTTCAAGCTTGGGAATAAAAGACAAACAAAGTTCTTGTTTGCTAAAAAAAAAAAAAAAAAAAAAAAAAAAA

>Butte_Gamma#4

CAAGAATCAAATCCAAGTAAGTAGTACCTAACGCAAATCCACCATGAAGACCTTACTCATCGTAACAATCCTTGCGATGGCAACAACCATCGCCACCGCCAATATGCAAGTCGACCCCGGCTACCAAGTACATTGGCCACAACAACAACCATTCCCCCAGCCCCAACAACCATTCTGCCAGCAACCACAACAAACTATTCCCCAACCCCATCAAACGTTCCACCATCAACCACAACAAACATTTCCCCAACCCCAACAAACATACCCCCATCAACCACAACAACAATTTCCCCAGACCCAGCAACCACAACAACCATTTCCCCAGCCCCAACAAACATTCCCCCAACAACCCCAACTACCATTTCCCCAACAACCCCAACAACCATTCCCCCAGCCTCAACAACCCCAACAACAATTTCCCCAGTCACAGCAACCACAACAACCTTTTCCCCAGCCCCAACAACAATTCCTGCAGCCCCAACAACCGCAACAATCATTCCCCCAGCAACAACAACCGTTGATTCAGCTATCTCTACAACAACAGATGAACCCCTGCAAGAATTTTCTCTTGCAGCAATGCAACCCTGTGTCATTGGTGTCATCCCTCATATCAATGATCTTGCCACGAAGTGATTGCCAGGTGATGCAGCAACAATGTTGCCAACAACTGGCACAGATTCCTCAGCAGCTCCAGTGTGCAGCCATCCATAGTGTCGTGCATTCCATCATCATGCAGCAAGAACAACGACAAGGCGTGCAGATCCGGCGGCCACTGTTTCAGCTCGTTCAGGGTCAGGGCATCATCCAACCTCAACAACCAGCTCAATTGGAGGTGATCAGGTCATTGGTATTGAGAACTCTTCCAACCATGTGCAACGTGTATGTCTCACCTGACTGCTCCACCATCAACGCACCATTTGCCAGCATAGTCGTCGGCATTGGTGGCCAATGAAAAATGCAAGAGTTATGCTAATAGGTAGATGGATCATCGTTGCTTAGCTGATGCACCAATCGTTGTAGCGATGACAAATAAAGTGGCGTACGCCATCATGTGTGACCCCGACCAGTGCTAGTTCAAGCTTGGGAATAAAAGACAAACAAAGTTCTTGTTTGCTAAAAAAAAAAAAAAAAAAAAA

>Butte_Gamma#5

GCACAAATCAGCAAATCCAAGTACGTAGTAGTTAACGCAAATCGACCATGAAGACCTTACTCATCCTGACGATCCTTGCGATGGCAATAACCATCAGCACCGCCAATATGCAGGTCGACCCTAGTGGCCAAGTACAATGGCCACAACAACAACTAGTCCCCCAACCCCAACAGCCATTATCCCAGCAACCGCAACAAGCATTTCCCCAACCCCAACAAACATTTCCCCATCAACCACAACAACAAGTTCCCCAGCCTCAGCAACCACAACAACCATTTCTCCAGCCCCAACAAGCATTCCCCCAACAACCACAACAACCATTCCCTCAGACTCAACAACCACAACAACCATTTCCCCAGCAACCACAACAACCATTCCCCCAGACTCAACAGCCACAACAACCATTTCCCCAGCAACCACAACAACCATTTCCCCAGCAACCACAACAACCATTCCCCCAGACTCAACAACCACAACAACCATTTCCCCAGCAACCACAACAACCATTCCCCCAGACTCAACAACCACAACAACCATTTCCCCAGTTCCAGCAACCACACCAACCTTTTCACCAGCCCCAACAACAATTCCCGCAGCCCCAACAACCGCAACAATCATTCCCCCAGCAACAACGACCGTTCATTCAGCCATCTCTACAACAACGTTTGAACCCATGCAAGAATATCCTCTTGCAACAATGCAAACCTGCGTCATTGGTGTCATCCCTCTGGTCGATAATCTGGCCACAAAGCGATTGCCAAGTGATGCAGCAACAATGCTGCCAAGAACTAGCACAGATTCCTCAGCAGCTCCAGTGCGCAGCCATCCATAGCGTCGTGCATTCCATCATCGTGCAGCAGCAACAACAACAACAACAACAACAACAACAACAACAACAAGGCATGCATATCCTGCTGCCACTATCTCAACAACAACAGTTGGGTCAAGGTACTCTCGTCCAAGGCCAGGGCATCATCCAACCTCAACAACTAGCTCAATTGGAGGCGATCAGGTCATTGGTGTTGCAAACTCTTCCAACCATGTGCAACGTGTATGTCCCACCTGAGTGCTCCATCATCAGGGCACCATTTGCCAGCATAGTCGCGGGGATTGGTGGCCAATGAAAAAAGCAAGATCTATACTAATAGATAGATGGATCATCGTTGCTTAGCAGATGCACCAATCGATGTAACGATGACAAATAAAGTGGCGTGCACCATCATGTGTGACCCCGACCAGTGCTAGTTCAAGCTTGGGAATAAAAGACAAACAGAGTTCTTGTTTGCCAGCATTGCTTGTCAAAAAAAAAAAAAAAA

>Butte_Gamma#6

TCCAATTAAGTAGTAGCCAACACAAATCCACCATGAAGACCTTATTCATCCTAACAATCCTTGCGATGGCAACAACTATCGCCACCGCGAATATGCAGGTCGACCCCAGCGGCCAAGTACAATGGCCACAACAACAACCATTCCGCCAGCCCCAACAACCATTCTACCAGCAACCACAACAAACATTTCCCCAACCCCAACAAGCATTCCCCCATCAACCACAACAACAATTTCCCCAGCCCCAGCAACCACAACAACAATTTCCGCAGCCCCAGCAACCACAACAACCATTTCCCCAGCCCCAACAAGCCCAACTACCATTTCCCCAACAACCACAACAACCATTCCCCCAGCCTCAACAACCCCAACAACCATTTCCCCAGTCACAGCAACCACAACAACCTTTTCCCCAGCCCCAACAACCGCAACAATCATTCCCCCAGCAACAACAACCGTTGATTCAGCCATATCTACAACAACAGATGAACCCTTGCAAGAATTACCTCTTACAGCAATGCAACCCTGTGTCATTGGTGTCATCCCTCGTGTCAATGATCTTGCCACGAAGTGATTGCCAGGTGATGCAGCAACAATGTTGCCAACAACTAGCACAGATTCCTCGCCAGCTCCAGTGTGCAGCCATCCATAGCGTCGTGCATTCCATCGTCATGCAGCAAGAACAACAACAAGGCATACAGATCCTCCGGCCACTGTTTCAGCTCGTCCAAGGTCAGGGCATCATCCAACCTCAACAACCAGCTCAATATGAGGTGATCAGATCATTGGTATTGAGAACCCTTCCAAACATGTGCAACGTGTATGTCCGACCTGACTGCTCCACCATCAACGCACCATTTGCCAGCATAGTCGCCGGCATCAGTGGACAATGAAAAATGAAGAGTTATGCTAATAGGTAGATGGATCATCGTTGCTTAGCTGATGCACCAGTCTTTGTAGCGATGAGGAATAAAGTGGCGTGCACCATCATGTGTGACCCCGACCAGAGCTAGTTCAAGCTTCAGAATAAAACACAAACAAAGTTCTTGTTTGCTAAAAAAAAAAAAAAAAAAAAA

>Butte_Gamma#7

CACAAGCATCAAATCCAAGTAGGTAGTAGTTAACGCAAATCCACCATGAAGACCTTACTCATCCTGACAATCCTTGCGATGGCAATAACCATCGCCACCGCCAATATGCAGGTCGACCCTAGCGGCCAAGTACAATGGCCGCAACAACAACCATTCCTGCAGCCTCACCAACCATTCTCCCAGCAACCACAACAAATATTTCCCCAACCCCAACAAACATTCCCCCATCAACCACAACAACAATTTCCCCAGCCTCAGCAACCACAACAACAATTTCTCCAGCCCCGACAACCATTCCCCCAACAACCACAACAACCATATCCCCAGCAACCACAACAACCGTTCCCCCAGACTCAACAACCCCAACAACCATTTCCCCAGTCCAAGCAACCACAACAACCTTTTCCCCAGCCCCAACAACCGCAACAATCATTCCCCCAACAACAACCATCGTTGATTCAACAATCTCTACAACAACAGTTGAACCCATGCAAGAATTTCCTCTTGCAGCAATGCAAACCTGTGTCCTTGGTGTCATCCCTCTGGTCAATCATCTTGCCACCAAGCGATTGCCAGGTGATGCGGCAACAATGTTGTCAACAACTAGCACAAATTCCTCAGCAACTCCAGTGTGCAGCCATCCATAGCGTCGTGCATTCCATCATCATGCAGCAAGAACAACAAGAACAACTACAGGGTGTGCAAATCCTGGTGCCACTGTCTCAACAGCAACAAGTGGGTCAAGGTATTCTCGTCCAGGGTCAAGGCATCATCCAACCTCAACAACCAGCTCAATTGGAGGTGATCAGGTCATTGGTGTTGCAAACTCTTCCAACCATGTGCAACGTGTATGTCCCACCTTACTGCTCCACCATCAGGGCACCATTTGCTAGCATAGTCGCCAGCATTGGTGGCCAATGAGAAAAAAGCAAGAGCTATTCTAAAAGTTAGATGGCCATCGTTGCTTAGCTTATGCACCAATCGATGTAACGATGACAAATAAAGTGGCGTGCACCATCATGTGTGATCCTGACCAAGTGCTAGTTCAAGATTGGAAATAAAAGACAAACTCAGTCCTTGTTTGCCAAAAAAAAAAAAAAAAAAA

>Butte_Gamma#8

CACCATGAAGACCTTACTCATCCTAACAATCCTTGCGATGGCAACAACCATCGCCACCGCCAATATGCAAGTCGACCCCAGCGGCCAAGTACAATGGCCACAACAACAACCATTCCCCCAGCCCCAACAACCATTCTGCCAGCAACCACAACGAACTATTCCCCAACCCCATCAAACATTCCACCATCAACCACAACAAACATTTCCTCAACCCCAACAAACATTCCCCCATCAACCACAACAACAATTTCCCCAGCCCCAGCAACCACAACAACCATTTCCCCAGCAACCACAACAACAATTTCCCCAGCCCCAACAACCACAACAACCATTTCCCCAGCAACCACAACAACAATTTCCCCAGCCCCAACAACCACAACAACCATTTCCCCAGCCCCAACAACCCCAACTACCATTTCCGCAACAACCACAACAACCATTCCCCCAGCCTCAACAACCCCAACAACCATTTCCCCAGTTACAGCAACCACAACAACCTTTACCCCAGCCCCAACAACCGCAACAACCATTCCCCCAGCAACAACAACCATTGATTCAGCCATACCTACAACAACAGATGAACCCCTGCAAGAATTACCTCTTGCAACAATGCAACCCTGTGTCATTGGTGTCATCCCTCGTGTCAATGATCTTGCCACGAAGTGATTGCAAGGTGATGCGGCAACAATGTTGCCAACAACTAGCACAGATTCCTCAGCAGCTCCAGTGCGCAGCCATCCATGGCGTCGTGCATTCCATCATCATGCAGCAAGAACAACAACAACAACAACAACAACAACAAGGCATACAGATCATGCGGCCACTATTTCAGCTCGTCCAGGGTCAGGGCATCATCCAACCTCAACAACCAGCTCAATTGGAGGTGATCAGGTCATTGGTATTGGGAACTCTTCCAACCATGTGCAACGTGTTTGTTCCACCTGAGTGCTCCACCACCAAGGCACCATTTGCCAGCATAGTCGCCGACATTGGTGGCCAATGAAAAATGCAAGCGTTATGCTAATAGGTAGATGGATCATCGTTGCTTAGTTGATGCACCAATCGTTGTAACGATGAAAAATAAAGTGGTGTGCACCATCATGTGTGA

>Butte_Gamma#9

TAAGTAGTATTTAACGAAAATCCACCATGAAGACCTTACTCATCCTAACAATCCTTGCGATGGCAACAACAATCGCCACTGCCAATATGCAGGTCGACCCTAGCAGCCGAGTACAATGGCCACAAGAACAACCACCCCCCCAGTCCCAACAACCATTCTCCCAGCAACCACAACAAATATTTCCCCAACCCCAACAAACATTCCCCCATCAACCACAACAAGCATTTCTCCAACCTCAACAAACATTCCCCCGTCGACCACAACAACAATTTCCCCAGCCCCAGCAACCACAACAACCATTTCCTCAGCCCCAACAACCCCAACTACCATTTCCCCAACAACCACAACAACCATTCCCCCAGCCTCAACAACCCCAACAACCATTTCCCCAGTCACAGCAACCACAACAACCTTTTCCCCAGCCCCAACAACAATTTCCGCAGCCCCAACAACCACAACAATCATTCCCCCAACAACAACAATGGATGATTCAGTCATTTCTACAACAACAGATGAACCCCTGCAAGAATTTCCTCTTGCAGCAATGCAACCCTGTGTCATTGGTGTCATCTCTCGTGTCAATAATCTTGCCACGAAGTGATTGCCAGCTGATGCAGCAACAATGTTGCCAACAACTAGCACAAATTCCTCAACAACTCCAGTGCGCAGCCATCCACAACGTCGCGCATTCCATCATCATGCAGCAAGAACAACAACGAGGCGTGCAGATCCTGCGGCCACTATTTCAGCTCGCCCAGGGTCTGGGTATCATCCAACCTCAACAACCAGCTCAATTGGAGGGGATCAGGCCATTGGTATTGAAAACTCTTCCAACCATGTGCAACGTGTATGTCCCACCTGACTGCTCCACCATCAACGTGCCATATGCCAGC

>Butte_Gamma#10

GTAAGTATTAGTTAACGCAAATCCACCATGAAGACCTTACTCATCCTAACAATCCTTGCGATGGCAACAACCATCGCCACCGCCAATATGCAAGTCGACCCCAGCGGCCAAGTACAATGGCCACAACAACAACCATTCCCCCAGCCCCAACAACCATTCTGCCAGCAACCACAACGAACTATTCCCCAACCCCATCAAACATTCCACCATCAACCACAACAATTTCCCCAGACCCAACAACCACAACAACCATTTCCCCAGCCCCAACAAACATTCCCCCAACAACCCCAACTACCATTTCCCCAACAACCCCAACAACCATTCCCCCAGCCTCAGCAACCCCAACAACCATTTCCCCAGTCACAACAACCACAACAACCTTTTCCCCAGCCCCAACAACAATTTCCGCAGCCCCAACAACCACAACAATCATTCCCCCAACAACAACAACCGGCGATTCAGTCATTTCTACAACAACAGATGAACCCCTGCAAGAATTTCCTCTTGCAGCAATGCAACCATGTGTCATTGGTGTCATCTCTCGTGTCAATAATTTTGCCACGAAGTGATTGCCAGGTGATGCAGCAACAATGTTGCCAACAACTAGCACAAATTCCTCAACAGCTCCAGTGCGCAGCCATCCACAGCGTCGCGCATTCCATCATCATGCAACAAGAACAACAACAAGGCGTGACGATCCTGCGGCCACTATTTCAGCTCGCCCAGGGTCTGGGTATCATCCAACCTCAACAACCAGCTCAATTGGAGGGGATCAGGTCATTGGTATTGAAAACTCTTCCAACCATGTGCAACGTGTATGTGCCACCTAACTGCTCCACCATCAACGTACCATATGCCAACATAGACGCTGGCATTGGTGGCCAATGAAAAATGCAAGATCATCGTTGCTTAGCTGATGCACCAATCGTTGTAGCGATGACAAATAAAGTGTGCACCATCATGTGTGACCCCGACCAGTGCTAGTTCAAGCTTGGGAATAAAAGACAAACAAAGTTCTTGTTTGCTAAAAAAAAAAAAAAAAAAAAAAAAAAAA

>Butte_Gamma#11

CCGCAACAATCATTCCCCCAACAACAACCACCGTTCATTCAGCCATCTCTACAACAACAGGTGAACCCATGCAAGAATTTCCTCTTGCAGCAATGCAAACCTGTGTCACTGGTGTCATCCCTCTGGTCAATGATCTGGCCACAAAGCGATTGCCAAGTGATGCGGCAACAATGCTGCCAACAACTAGCACAGATTCCTCAGCAGCTCCAGTGTGCAGCCATCCATACCGTCATACATTCCATCATCATGCAGCAAGAACAACAACAAGGCATGCATATCCTGCTGCCACTATATCAGCAGCAACAGGTGGGTCAAGGTACTCTCGTCCAGGGCCAGGGCATCATCCAACCCCAACAACCAGCTCAATTGGAGGCGATCAGGTCATTGGTGTTGCAAACTCTTCCAACCATGTGCAACGTGTATGTCCCACCTGAGTGCTCCATCATCAAGGCACCATTTTCCAGCGTAGTCGCCGGCATTGGTGGCCAATGAAAAATGCAAGAGCCATACTAATAGGTAGATGGATCATCGTTGCTTAGTTGATTTACCCATCGATGTAACGATGATAGAATAAACTGGCGTGCACCATCATGTGTGACCCCGACCAGTGCAATTTCAAGCTTGGGAATAAAAGACAAAGAAAGTTCAAGTTTGCGAAAAAAAAAAAAAAAAAAAA
