# Supplementary material for: Analysis of expressed sequence tags from a single wheat cultivar facilitates interpretation of tandem mass spectrometry data and discrimination of gamma gliadin proteins that may play different functional roles in flour
Source: BMC Plant Biol. 2010 Jan 11;10:7. doi: 10.1186/1471-2229-10-7 (PMC2827424; doi:10.1186/1471-2229-10-7)
Supplement: Additional file 3 — Amino acid sequences of gamma gliadins deduced from consensus sequences of Butte 86 contigs. Peptides identified by MS/MS after digestion with chymotrypsin are indicated in bold, thermolysin are underlined, and trypsin are enclosed in boxes. [file 1471-2229-10-7-S3.PDF]

Additional File 3.

>Gamma#1
MKTLLILTIIAVALTTTTANIQVDPSGQVQWPQQQPFPQPQQPQQIFPQPQ**QTFPHQP**
QQAFPQPQ**QTFPHQPQ**QQFPQPQQPQQPFPQQPQQQFPQPQQPQQPFPQQPQQQFPQPQ
QPQQPFPQPQQPQLPFPQQPQQPFPQPQQPQQPFPQLQQPQQPLPQPQQPQQPFPQQQQ
PLIQPYLQQQMNPCKNYLLQQCNPVSLVSSLVSMILPRSDCKVMRQQCCQQLAQIPQQL
QCAAIHGVVHSIIMQQEQQQQQQQQQGIQIMRPLFQLVQGQGIIQPQQPAQLEVIRSLV
LGTLPTMCNVFVPPECSTT**KAPFASIVADIGGQ**
>Gamma#2
MKTLLILTILAMAITIGTA**NIQVDPSGQVQWLQQQLVPQLQQPL**SQQPQQTFPQPQQTF
PHQPQQQVPQPQQPQQPFLQPQQPFPQQPQQPFPQTQQPQQPFPQQPQQPFPQTQQPQQ
PFPQQPQQPFPQTQQPQQPFPQLQQPQQPFPQPQ**QQLPQPQQPQQSFPQQQR**SFIQPSL
QQQLNPCKNILLQQCKPASLVSSLW**SIIWPQSDCQVM**RQQCCQQLAQIPQQLQCAAIHS
VVHSIIMQQQQQQQQQQGMHIFLPL**SQQQQVGQGSLVQGQGIIQPQQPAQLEAIRSLVL**
QTLPSMCNVYVPPECSIMRAPF**ASIVAGIGGQ**
>Gamma#3
MKTLLILTILAMATTIATANMQVDPSGQVQWPQQQPFPQPQQPFCQQPQRTIPQPHQTF
HHQPQQTFPQPQQTYPHQPQQQFPQTQQPQQPFPQPQQTFPQQPQLPFPQQPQQPFPQP
QQPQQPFPQSQQPQQPFPQPQQQFPQPQQPQQSFPQQQQPAIQSFLQQQMNPCKNFLLQ
QCNHVSLVSSLVSIILPRSDCQVMQQQCCQQLAQIPQQLQCAAIHSVAHSIIMQQEQQQ
GVTILRPL**FQLAQGLGIIQPQQPAQLEGIRSLVL**KTLPTMCNVY**VPPNCSTINVPYANI**
DAGIGGQ
>Gamma#4
MKTLLIVTILAMATTIATANMQVDPGYQVHWPQQQPFPQPQQPF**CQQPQQTIPQPHQTF**
HHQPQQTFPQPQQTYPHQPQQQFPQTQQPQQPFPQPQQTFPQQPQLPFPQQPQQPFPQP
QQPQQQFPQSQQPQQPFPQPQQQF**LQPQQPQQSFPQQQQPLIQLSL**QQQMNPCKNFLLQ
QCNPVSLVSSLISMILPRSDCQVMQQQCCQQLAQIPQQLQCAAIHSVVH**SIIMQQEQRQ**
GVQIRRPLFQLVQGQGIIQPQQPAQLEVIRSLVLRTLPTMCNVY**VSPDCSTINAPFASI**
VVGIGGQ
>Gamma#5
MKTLLILTILAMAITISTANMQVDPSGQVQWPQQQLVPQPQQPL**SQQPQQAFPQPQQTF**
PHQPQQQVPQPQQPQQPFLQPQQAFPQQPQQPFPQTQQPQQPFPQQPQQPFPQTQQPQQ
PFPQQPQQPFPQQPQQPFPQTQQPQQPFPQQPQQPFPQ**TQQPQQPFPQFQQPHQPFHQP**
QQQFPQPQQPQQSFPQQQRPFIQPSLQQRLNPCKNILLQQCKPASLVSSLWSIIWPQSD
CQVMQQQCCQELAQIPQQLQCAAIHSVVHSIIVQQQQQQQQQQQQQQQGMH**ILLPLSQQ**
QQLGQGTLVQGQGIIQPQQLAQLEAIRSLVLQTLPTMCN**VYVPPECSIIRAPFASIVAG**
IGGQ
>Gamma#6
MKTLFILTILAMATTIATANMQVDPSGQVQWPQQQPF**RQPQQPFYQQPQQTFPQPQ**QAF
PHQPQQQFPQPQQPQQQFPQPQQPQQPFPQPQQAQLPFPQQPQQPFPQPQQPQQPFPQS
QQPQQPFPQPQQPQQSFPQQQQPLIQPYLQQQMNPCKNYLLQQCNPVSLVSSLVSMILP
RSDCQVMQQQCCQQLAQIPRQLQCAAIHSVVHSIVMQQEQQQGIQILRPLFQLVQGQGI
IQPQQPAQY**EVIRSLVL**RTLPNMCNVYVRPDCSTINAPF**ASIVAGISGQ**
>Gamma#7
MKTLLILTILAMAITIATANMQVDPSGQVQWPQQQPF**LQPHQPF**SQQPQQIFPQPQQTF
PHQPQQQFPQPQQPQQQFLQPRQPFPQQPQQPYPQQPQQPFPQTQQPQQPFPQSKQPQQ
PFPQPQQPQQSFPQQQPSLIQQSLQQQLNPCKNFLLQQCKPVSLVSSLWSIILPPSDCQ

VMRQQCCQQLAQIPQQLQCAAIHSVVHSIIMQQEQQEQL**QGVQILVPLSQQQQVGQGIL**
VQGQGIIQPQQPAQLEVIRSLVLQTLPTMCNVYVPPYCSTIRAPF**ASIVASIGGQ**
>Gamma#8
MKTLLILTILAMATTIATANMQVDPSGQVQWPQQQPFPQPQQPFCQQPQRTIPQPHQTF
HHQPQQTFPQPQ**QTFPHQPQ**QQFPQPQQPQQPFPQQPQQQFPQPQQPQQPFPQQPQQQF
PQPQQPQQPFPQPQQPQLPFPQQPQQPFPQPQQPQQPFPQLQQPQQPLPQPQQPQQPFP
QQQQPLIQPYLQQQMNPCKNYLLQQCNPVSLVSSLVSMILPRSDCKVMRQQCCQQLAQI
PQQLQCAAIHGVVHSIIMQQEQQQQQQQQQGIQIMRPLFQLVQGQGIIQPQQPAQLEVI
RSLVLGTLPTMCNVFVPPECSTT**KAPFASIVADIGGQ**
>Gamma#9
MKTLLILTILAMATTIATANMQVDPSSRVQWPQEQPPPQSQQPFSQQPQQIFPQPQQTF
PHQPQQAFLQPQQTFPRRPQQQFPQPQQPQQPFPQPQQPQLPFPQQPQQPFPQPQQPQQ
PFPQSQQPQQPFPQPQQQFPQPQQPQQSFPQQQQWMIQSFLQQQMNPCKNFLLQQCNPV
SLVSSLVSIILPRSDCQLMQQQCCQQLAQIPQQLQCAAIHNVAHSIIMQQEQQRGVQIL
RPLFQLAQGLGIIQPQQPAQLEGIRPLVLKTLPTMCNVYVPPDCSTINVPYAS
>Gamma#10
MKTLLILTILAMATTIATANMQVDPSGQVQWPQQQPFPQPQQPFCQQPQRTIPQPHQTF
HHQPQQFPQTQQPQQPFPQPQQTFPQQPQLPFPQQPQQPFPQPQQPQQPFPQSQQPQQP
FPQPQQQFPQPQQPQQSFPQQQQPAIQSFLQQQMNPCKNFLLQQCNHVSLVSSLVSIIL
PRSDCQVMQQQCCQQLAQIPQQLQCAAIHSVAHSIIMQQEQQQGVTILRPL**FQLAQGLG**
IIQPQQPAQLEGIRSLVLKTLPTMCNVY**VPPNCSTINVPYANIDAGIGGQ**
>Gamma#11
PQQSFPQQQPPFIQPSLQQQVNPCKNFLLQQCKPVSLVSSLWSMIWPQSDCQVMRQQCC
QQLAQIPQQLQCAAIHTVIHSIIMQQEQQQGMHILLPL**YQQQQVGQGTLVQGQGIIQPQ**
QPAQLEAIRSLVLQTLPTMCNVYVPPECSIIKAPF**SSVVAGIGGQ**
